# History, Studies and Specific Uses of Repetitive Transcranial Magnetic Stimulation (rTMS) in Treating Epilepsy

**Published:** 2016

**Authors:** Sima NOOHI, Susan AMIRSALARI

**Affiliations:** 1 Psychiatrist, Behavioral Sciences Rsearch Center, Baqiyattalah University of Mecial Sciences, Tehran, Iran; 2 Pediatric Neurologist, New Hearing Technologies Research Center, Baqiyatallah University of Medical Sciences, Tehran, Iran.

**Keywords:** Repetitive Transcranial Magnetic Stimulation (rTMS), Epilepsy, Treatment

## Abstract

**Objective:**

In this study, repetitive Transcranial Magnetic Stimulation (rTMS) and its specific use for treating epilepsy were carefully scrutinized.

**Materials & Methods:**

Target researches such as review articles, case reports, books and theses, which had to do with therapeutic method of rTMS were surveyed. It is worth mentioning that until the final stages, the search for records and documents related to rTMS went on and in the end, the collected data underwent a qualitative analysis.

**Results:**

As the literature review suggests, TMS principally applies electromagnetic induction to generate an electric current inside the brain without physical contact. The therapeutic uses of rTMS are for a wide range of mental disorders, namely epilepsy, chronic pains, motor disorders and so on.

**Conclusion:**

Despite safety concerns and possible side effects, many researchers subscribe to rTMS and see a bright future for it.

## Introduction

**Fig 1:**
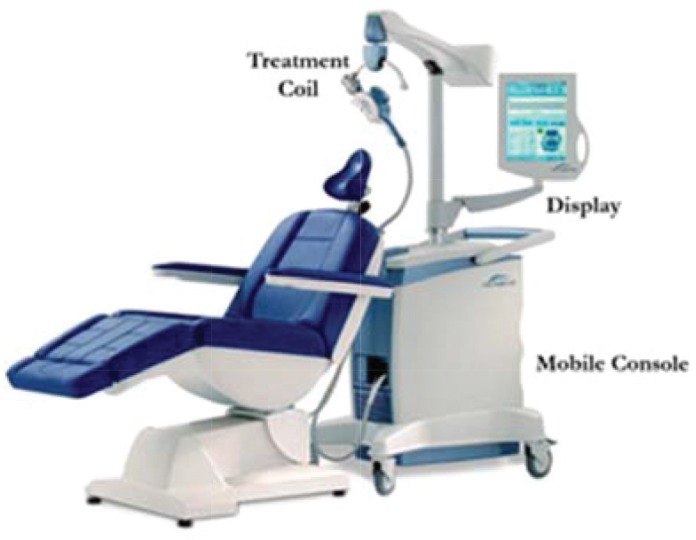
rTMS Unit

**Fig 2:**
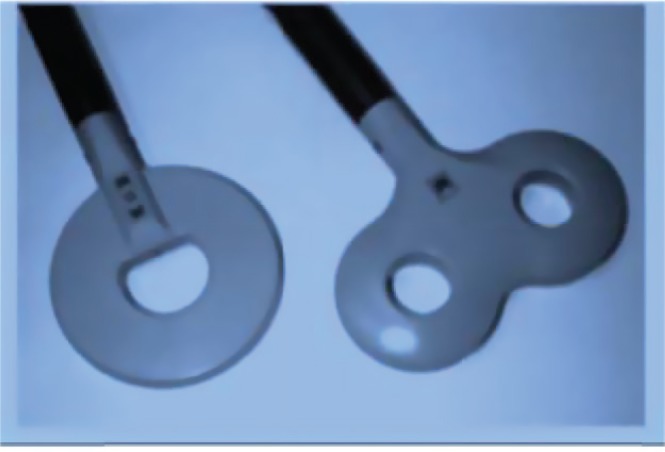
rTMS coils

Over the recent decades, electrophysiological studies have much broadened the understanding of brain’s normal activity and its pathological conditions. Electrophysiology has also made useful contributions to successful therapeutic plans. Technological advances have played a major role in either attaining therapeutic goals or doing cutting-edge researches in a variety of disciplines like neurology, psychiatry and psychology. One of the impressive achievements of electrophysiology seems to be “brain stimulation” and technologies thereof. Higgins and George (1) argue that a great volume of research in fundamental and clinical areas is being conducted on the methods of brain stimulation. Although methods of brain stimulation have come with favorable results, their side effects are of considerable concern. Accordingly, researchers strive to explore and develop new ways with a lower level of risk. Exponents of brain stimulation always seek less invasive methods, that is, they make any effort to achieve significant outcomes by focal stimulation of cortex areas without imposing a vast convulsion (2). These efforts have brought forth modern methods of brain stimulation such as Vagus Nerve Stimulation (VNS), Transcranial Electrical Stimulation (TES), Transcranial Alternating Current Stimulation (TACS), Transcranial Direct Current Stimulation (TDCS), Highdefinition Transcranial Direct Current Stimulation (HD-TDCS), Low-field Magnetic Stimulation (LFMS), Transcranial Random Noise Stimulation (TRNS), Cranial Electric Stimulation (CES), Magnetic Seizure Therapy (MST) and Deep Brain Stimulation (DBS). From the new methods mentioned above, “Transcranial Magnetic Stimulation” (TMS) is the one, which has recently acquired a great deal of reputation (Figure 1). TMS was first introduced by Barker et al., (3). Briefly, TMS is the induction of an electric current to the brain cortex through a magnetic field outside the brain (4). Repetitive TMS (rTMS) is a variation of TMS where stimulation is provided in sessions of same condition to create long-term excitation in the brain cortex (5). In rTMS, the electric activities in the brain are influenced by the magnetic fields. The electromagnetic induction generated by rTMS is painless and safely passes through the skin and skull. Then, it produces small electric currents in the region of the brain just under the coil (Figure 2). In this procedure of treatment, multiple parameters are involved that can be changed to reach different treatment goals. These concurrent parameters include the number of stimulations, stimulation intensity, stimulation frequency, length of intervals between stimulations and the stimulated areas of the brain (6). Kaplan et al., have categorized uses of brain stimulation into three areas, namely pathophysiology, brain’s normal functions and treatment (7). While the modern technology of TMS makes its way forward, a growing number of studies on its applications are done. Subsequently, these attempts entail undertaking a comprehensive review to lay appropriate foundations for further fundamental and applied researches. Hence, the present study aimed to review the history, researches and specific uses of rTMS for the treatment of epilepsy. In this regard, the whole literature on TMS came under rigorous scrutiny thereby gaining a comprehensive understanding of theories, researches and uses of rTMS.

**History of Transcranial Magnetic Stimulation**

TMS consists in the law of electromagnetic induction, a process in which electrical energy converts to magnetic energy and vice versa. From a historic point of view, first it was Faraday, in 1831, arguing that the relation between electrical energy and magnetic field was reciprocal (8). Later, further experiments revealed that the use of magnetic coils over a person’s head could bring about feelings of dizziness and giddiness. Attempts were also made to produce vibration of the skull through electromagnetic coils placed on the head of depressed patients. In fact, the ultimate goal was stimulation of the nerve cells of human (9). Later, magnetic coils were used to treat mental disorders of depression and neurosis via electrical effects of the magnetic field created on the scalp (9). A defect that most of early experiments suffered from was that the area of the skull placed beneath the coil was not clearly described (6). On the other hand, the capacitors were not capable of generating high intensity or fast frequency stimulations either. As a result, how remissions took place at the time is now in total obscurity. The current use of electromagnetic induction for transcranial stimulation dates back to 1985. At that time, Barker and his colleagues invented the initial type of TMS in Sheffield, UK (3). Moreover, Barker scientifically proved the influence of magnetic stimulation on motor cortex of the human brain in 1985. However, the original tools were partly slow when recharging and repetitive uses could highly raise the coil’s temperature. Their workplace is still in operation developing novel tools and devices. Nowadays, advances in technology have made it possible for us to manipulate the procedure of transcranial magnetic stimulation in either form of ceaseless or repetitive pulses over any part of the cortex. The form of repetitive stimulation is abbreviated to rTMS (10). The first use of rTMS was restricted to diagnosis of neuro-motor disorders. Several years before, scientists suggested that the application of rTMS on cortex regions brought about effects similar to antidepressants. However, the effects of TMS on prefrontal area did not draw attentions early in the way of TMS development. In 2002, Canadian Association of Health which is an official institution approved the medical results and benefits of rTMS. Moreover, other countries like the US, the UK, Germany and Japan have been working on this method for 20 years. At present, devices for repetitive electromagnetic induction have been developed into a structure that different parameters, e.g. the ability to increase or decrease the magnetic energy on cortical areas, can be precisely modified to fit best the treatment of various psychological disorders. Food and Drug administration of the United States also granted approval to this method on October 8th, 2008 (6).

**How Transcranial Magnetic Stimulation works**

Different parts of TMS mechanism from the pulse inside the coil to the intensity of the stimulation of the nerve cell, altogether obey several laws of physics. Generally, TMS equipment is simple and consists of a transformer for charging a big capacitor, which instantly discharges to create a magnetic field pulse in the coil for stimulation. Actually, there is a sub-circuit to control the temperature, intensity and repetition of the pulse. The maximum voltage approximately equals 2000 volts and generated currents come nearly to 10000 Amps. It is necessary to have a high-voltage electrical switch to create a short pulse (nearly 250 microseconds or 1/4000 of a second) for effective stimulation (11). A high-tension cord is also necessary for connecting the coil to the head to transfer effectively the strong current. In the TMS mechanism, when the device is charged with electrical current, magnetic fields occur around the coils. To be precise, according to the Faraday’s Law, if there are two coils beside each other, one charged with electricity and another devoid of electrical energy, as the current in the first coil stops, a very short pulse is transferred to the second coil. In a technical word, the second coil is not a necessary factor through this process and the current can be transferred to any other conductor close to the first coil, whether it is the brain or a coil (12). Repetitive TMS also goes the same way as above. In this type of treatment, the magnetic field produced by the first coil is transferred to the second, meaning the brain, to stimulate the nerve cells located there. Actually, when the magnetic fields go through the brain, they generate a secondary electric current, which changes the electrical load of the nerve cells. It is notable that existing equipment of rTMS is capable of producing magnetic induction as deep as two centimeters into the brain. This technology can easily excite regions between the white matter and the gray matter of the brain. On this account, the neural axons transfer the generated currents close to two centimeters beneath the coil. In addition, the electric current, which causes change of neuro-activities is in the vicinity of 70 millivolts (12).

**Uses of rTMS in the treatment of epilepsy**

Epilepsy is the most prevalent neural chronic disorder and more than 40 million people suffer from the disorder worldwide. This disease is characterized by different types of relapsing electrographic seizures. Such attacks are rooted in the brain starting from the center then gradually develop toward other parts of the brain or the whole organ. Although there are different types of epilepsy, they all have a common physiological characteristic. The excitability of the cortex increases in epilepsy cases (13). Epileptic attacks are the consequences of imbalance in prevention and neural communication stimulation, which are caused by sudden and uncontrolled discharge of neurons in the central neural system (14). Transcranial magnetic stimulation has been used to decrease and prevent epileptic seizures. In addition, there has been a multitude of researches done using TMS to probe the pathophysiology of the disease. The lowest level of stimulation, in which single TMS pulses cause distinguishable muscular activities, is called Motor Threshold (MT). MT is usually used to adjust the intensity of TMS for other applications or for estimating its safety (15). Since single TMS pulses seem to induce motor activities, it can be assumed that a chain of such pulses would have the same activating result. This hypothesis was assumed true in the preliminary studies of the effects of TMS on epilepsy. However, some researches have shown, long strings of pulses with 1Hz or less intension have preventive effects on sensory, locomotive and visual areas (16, 17). Moreover, there is increasing evidence that shows TMS has non-stimulating effects. Most of such effects are preventive, but some others can bring about stimulation. It is worth saying that repetitive TMS higher than 1Hz of intension along with strings of high frequency waves could be stimulating (18). In a theoretical point of view, kindling is a potential threat of TMS. Kindling is a process, in which daily harmless stimulation of the cortex leads to epileptic attacks or unstudied epileptic convulsion (9). In comparison with other parts of the brain, hippocampus and amygdala are kindled easier in rodents. The cortex is more resistant to kindling than the limbic system. In addition, forecerebrum regions are more resistant to kindling in smaller animals. Preliminary experiences of kindling were performed using pulses, which were emitted from electrodes implanted in the gray matter. Kindling is a standard model for the study of the effects of different treatment factors on epilepsy and its consequent convulsions. It is known that chemical drugs significantly affect behavioral and electrophysiological facets of convulsion in kindling model. Giving such medication along with application of a 50_Hz magnetic field, have indicated little preventive effects on the process of convulsion by kindling. The first research for the investigation of the medical effects of kindling with frequencies of 0.1, 0.5, 1 and 2Hz rTMS, in optimized spatial dimensions for the coil and following high standards of kindling model, was conducted over the area of amygdale by Yadollahpoor et al., (13). They also investigated the effects of the intensity of magnetic field by applying 80%, 90% and 100% of the field power on the motor threshold. The kindling process and its consequent convulsion are very likely to occur with the frequencies close to 60_ Hz while it is unlikely to happen with 10Hz or lower frequencies. Thus, the probable relationship between kindling and long-term empowerment as well as its effect on human epilepsy are worthy of consideration regardless of some divergent results (9). Although 1_Hz stimulations of the inside of the skull by electrodes have shown kindling phenomenon, the string of pulses with such a frequency or lower than this, mostly result in long term weakening of synaptic transfer. These findings along with many other findings are in line with the preventive effect of TMS (18). The results indicate that the excitability of the cortex can be adjusted by strings of repeated magnetic pulses (10, 19). Repetitive TMS allows for the activation of cortex areas. Activating strings with different frequencies create preventive effects and stimulation both at the time of using and after that. It seems that preventive effects of TMS are useful for reducing the excitability of neurons. Given the huge number of researches about the anti-convulsive effects of rTMS, there is still no agreement on an optimized pattern of rTMS (20). In other words, the patterns of rTMS application to achieve the maximum efficiency are still unclear. Frequency of rTMS is one of the most important factors. Additionally, the duration of its application can have a significant role in its effectiveness (21). Low frequency rTMS, too, can have therapeutic effects on epilepsy (22). Menkes and Gruenthal indicated that rTMS with low frequencies (0.3 and 0.5Hz) could yield therapeutic results as to epilepsy (23). They believe that using this technique with low frequencies results in the relief of neural activity and reduction of cortex excitability. In some experiences on epileptic patients resistant to medical treatment, even high frequencies could reduce the frequency of epileptic attacks (24). Different parameters of stimulation (such as frequency, intensity of the field, the duration of the stimulation, etc.) had not been well taken into consideration. Currently, anti-convulsion drugs and in some cases surgery are employed to treat epileptic attacks. Each of these methods has side effects of its own kind. Moreover, some types of epilepsy are resistant to medical treatment and surgery is not an option. Repetitive TMS can have high safety due to its non-invasive and painless procedure. In addition, its low cost and harmless side effects are some advantages of this technique. The attempts for acquiring optimized parameters including frequency, intensity, duration, and spatial factors of coil location can result in valuable niches for the replacement or integration of this method with common methods of epilepsy treatment (which have many side effects) (13). Contrary to electrical stimulation wherein the location of stimulation conforms to a number specifications, the best practice for the location of stimulation in rTMS method have not yet developed and we are still making attempts to focalize the stimulations (13). The results indicate that 1_Hz frequency with 80% and 90% of motor threshold has significant preventive effects on the extension of stimulation to other areas of the cortex. Raising the intensity from 80% to 90%, could increase the preventive effects of this technique. However, this increase showed an unvarying level of preventive effect while the increase from 90% to 100% decreased the preventive effect. Accordingly, they concluded that the frequency of rTMS stimulation and the motor threshold of the animal have significant roles in creating physiological effects. According to the findings, rTMS technique has positive effects on epileptic patients with specific frequencies and intensities. The researchers have attempted to administer low frequency rTMS for the treatment of epileptic disorders as well as other excessive excitabilities of the cortex. The potential of using rTMS for the treatment of epilepsy has been proposed recently, because rTMS has preventive effects on motor cortex (16). Studies show the effectiveness of low frequency rTMS for reducing the excitability of the cortex in patients with drug-resistant epileptic attacks. Such studies show more recovery in patients with focal lesion in comparison to patients with lesion in several areas. Some scientists with the help of some laboratory experiences showed that low frequency electrical stimulation prevents convulsive attacks (25). Steinhoff et al., for the first time reported the application of low frequency rTMS on seven patients with focal epilepsy of metro-cortical area of the limbic system (26). Magnetic stimulation of the lower frontal cortex showed that there is more reduction of electroencephalogram waves in diverted stimulation. Low frequency rTMS was applied on 9 patients with uncontrolled focal epilepsy with medical treatment in another study. The results showed 39% reduction of convulsive attacks for 4 weeks after the end of stimulations. The convulsive attacks returned to their previous rate 6-8 weeks later (27). Kinoshita et al., investigated the effects of the application of rTMS on the reduction of convulsive attacks in patients with extra-temporal epilepsy (28). Six patients with drugresistant epilepsy were treated with 0.9_Hz rTMS for 15 min a day in five days of the week with the intensity of 90%. The number of medications was calculated two weeks before and after rTMS. In addition, the resting motor threshold and the active motor threshold were checked before and after the treatment every day. The occurrence of both complex partial and simple partial epilepsy was 19.1% and 35.7%, reduced after one week of treatment with rTMS. Misawa et al. studied the effects of low frequency rTMS on the epilepsy caused by cortical dysplasia (29). A 31 yr old epileptic patient was investigated in terms of upper and lower extremities. Cortical dysplasia was located in motor cortex. Low frequency rTMS of the arm was performed with 90% of the motor threshold and 0.5_ Hz frequency. The results showed the reduction of high excitability after the application of rTMS. Thus, low frequency rTMS can prevent the excitability of the cortex and can be used as an effective treatment for local epilepsy.

## Discussion

At present, it seems necessary to conduct further studies to understand if there is any specific disorder responding well to rTMS. In addition, there are still several questions unanswered such as how long the antidepressant effect of rTMS remains, if medications play roles in combination with rTMS, and whether rTMS can solely be administered during acute treatment. Another question is that, after the use of rTMS in acute treatment, if it acts as a preventive agent. Apart from safety concerns, the risk-benefit ratio of this method falls under the influence of subjects’ personal differences, different operating ways plus the purpose that the device is used for. To tell the truth, such these parameters should be set into globally accepted standards both for clinical uses and for accreditation of rTMS physicians. In other words, these standards specify the prerequisites of operating rTMS method and clarify the best practice to work rTMS. Currently, there is no detailed compilation of training instructions for rTMS. Nonetheless, it is highly recommended that physicians expand their knowledge of physiological laws of the brain and rTMS mechanism of action, technical information, protocols and safety provisions. The need for rigorous guidelines and instructions is really urgent. On the other hand, if a treatment model is extracted out of research findings, the procedure may contain a few flaws, that is to say, it is not always feasible to generalize the effects of rTMS on a specific sample population to other clinical patients (9). The fundamental concern which most researchers express about rTMS is that how long the therapeutic effects of rTMS last and from a medical standpoint, whether it suffices. There are two answers to this question. First, in some cases if a long-term treatment is available later in time, a provisional effect seems enough then. Besides, administration of rTMS has proved to be useful particularly for those who suffer from drugresistant disorders too. The second answer is borrowed from the expectation about medical results of rTMS. According to a remedial model, rTMS is to improve functional imbalance of a patient. So if the effects are temporary, the imbalance recurs and the symptoms reappear as a result. In such a situation, rTMS as an effective method is believed to create permanent effects on the brain circuit. However, there is no evidence to prove such a thing at the moment. All things considered, rTMS is capable of producing a combination of stimulating and preventive effects, contrary to the best drugs that just have a few pharmaceutical benefits. Therefore, it is not unlikely that in the near future, rTMS could treat the lost function of a patient with Parkinson as dopamine-therapy does. Repetitive TMS will be able to reach different therapeutic goals through the manipulation of electrophysiological parameters (30). Repetitive TMS should be developed in a way so that it changes into a mixed treatment model which takes place together with other therapeutic methods to get the optimum effect. Such a model does not exclude other techniques of treatment, but rather it employs a blend of methods to deal with medical conditions. Above all, rTMS can help the brain to cope with conditions by itself. This idea comes from the studies of apoplexy, in which there is a common belief that some functional achievements regained after the stroke, originate from the ability of the brain to reorganize itself in normal routes. This ability partly results in the compensation of the lost functions belonged to other regions. To enhance this ability, some types of rTMS can play a supporting role thereby promoting the natural adaptation to the damage or chronic illness. Thus far, the use of rTMS as a therapeutic technique has accompanied by other methods of treatment, building a mixed model which outdo in the explanation of stable effects on symptoms. But it is a matter of regret that studies have little explored this combination. Besides, many studies include a few sample populations, referring just to one center, which makes it difficult to approve their study design and results. Nonetheless, there are many experiments in the effects of rTMS on depression. Reviewing the background of such attempts can help new comers, clinicians and researchers with the identification of existing flaws in rTMS method so that they could implement the best practice in the future (31). There are also clues that some cortical regions of the brain require different types of rTMS treatment. In this regard, investigation of parameters like intensity, quantity of pluses in each session and quantity of sessions seems important to gain better medical outcomes (32).

**In conclusion**, TMS has therapeutic uses for a wide range of mental disorders, namely epilepsy, chronic pains, motor disorders and so on. This method through electromagnetic induction generates an electric current inside the brain without physical contact. Although there might be concerns about safety and side effects of rTMS, many scholars and clinicians subscribe to this technology and see a bright future for it.

## References

[1] Higgins ES, George MS (2008). Brain Stimulation Therapies for the Clinician.

[2] Sackeim HA, Prudic J, Devanand DP, Nobler MS, Lisanby SH, Peyser S (2000). A prospective, randomized, double-blind comparison of bilateral and right unilateral electroconvulsive therapy at different stimulus intensities. Arch Gen Psychiatry.

[3] Barker AT, Jalinous R, Freeston IL Non-invasive magnetic stimulation of human motor cortex. Lancet.

[4] Wassermann EM, Zimmermann T (2012). Transcranial magnetic brain stimulation: therapeutic promises and scientific gaps. Pharmacol Therapeutics.

[5] Wassermann EM, Lisanby SH (2001). Therapeutic application of repetitive transcranial magnetic stimulation: a review. Clin Neurophysiol.

[6] Khomami S, Rostami R (2008). Remedy of Social Functions and Symptoms of Depression before and after Repetitive Stimulation (rTMS) on the Skull in Major Depression Patients [dissertation].

[7] Sadock BJ, Sadock VA, Ruiz P, Boutros N, Iacono W, Galderisi S (2009). Applied Electrophysiology. Kaplan and Sadock’s Comprehensive Textbook of Psychiatry.

[8] Marcolin MA, Padberg F, Kaschka WP, Gattaz WF (2007). Transcranial Brain Stimulation for Treatment of Psychiatric Disorders.

[9] George MS, Belmaker RH (2007). Transcranial Magnetic Stimulation in Neuropsychiatry.

[10] Pascual-Leone A, Valls-Sole J, Brasil-Neto JP, Cohen LG, Hallett M (1994). Akinesia in Parkinson’s disease. I. Shortening of simple reaction time with focal, single-pulse transcranial magnetic stimulation. Neurology.

[11] Roth BJ, Cohen LG, Hallett M (1991). The electric field induced during magnetic stimulation. Electroencephalogr and Clin Neurophysiol Suppl.

[12] Rezayi-Koochaksarayi M (2011). The Effect of Repetitive Magnetic Stimulation of the Skull (rTMS) on Drug Consumption and Psychological Well-being in Methamphetamine (crystal) Dependent Addicts [dissetation].

[13] Yadollahpour A, Firoozabadi SM, Mirnajafizade SJ (2015). Investigating the Anticonvulsant Effects of Repetitive Transcranial Magnetic Stimulation on Perforant Path Kindling Model in Rats. Zah J Res Med Sci.

[14] Abel MS, McCandless DW, Boulton AA, Butterworth RF (1992). Animal Models of Neurological Disease.

[15] Mongabadi S, Firouzabadi SMP, Mirnajafi-Zadeh J (2008). Effect of different frequencies of repetitive transcranial magnetic stimulation (rTMS) on acquisition of chemical kindling seizures in rats. Physiol Pharmacol.

[16] Chen R, Classen J, Gerloff C, Celnik P, Wassermann EM, Hallett M (1997). Depression of motor cortex excitability by low-frequency transcranial magnetic stimulation. Neurology.

[17] Knecht S, Ellger T, Breitenstein C, Bernd Ringelstein E, Henningsen H (2003). Changing cortical excitability with lowfrequency transcranial magnetic stimulation can induce sustained disruption of tactile perception. Biol Psychiatry.

[18] Wassermann EM (1998). Risk and safety of repetitive transcranial magnetic stimulation: report and suggested guidelines from the International Workshop on the Safety of Repetitive Transcranial Magnetic Stimulation, June 5-7,1996. Electroencephal Clin Neurophysiol.

[19] Maeda F, Keenan JP, Tormos JM, Topka H, Pascual-Leone A (2000). Modulation of corticospinal excitability by repetitive transcranial magnetic stimulation. Clin Neurophysiol.

[20] Post RM, Speer AM, Weiss SR, Li H (2000). Seizure models: anticonvulsant effects of ECT and rTMS. Prog in Neuropsychopharmacol Biol Psychiatry.

[21] Cincotta M, Borgheresi A, Gambetti C, Balestrieri F, Rossi L, Zaccara G (2003). Suprathreshold 0.3 Hz repetitive TMS prolongs the cortical silent period: potential implications for therapeutic trials in epilepsy. Clin Neurophysiol.

[22] Theodore WH, Hunter K, Chen R, Vega-Bermudez F, Boroojerdi B, Reeves-Tyer P (2002). Transcranial magnetic stimulation for the treatment of seizures: a controlled study. Neurology.

[23] Menkes DL, Gruenthal M (2000). Slow-frequency repetitive transcranial magnetic stimulation in a patient with focal cortical dysplasia. Epilepsia.

[24] Akamatsu N, Yukiko F, Yutaka E, Akira T, Tomonari Y, Takenori U (2005). The therapeutic effects of highfrequency transcranial magnetic stimulation on pentylenetetrazol-induced status epilepticus in rats. Inter Cong Ser.

[25] Hoogendam JM, Ramakers GM, Di Lazzaro V (2010). Physiology of repetitive transcranial magnetic stimulation of the human brain. Brain Stimul.

[26] Steinhoff BJ, Stodieck SR, Paulus W, Witt TN (1992). Transcranial stimulation. Neurology.

[27] Kanai R, Chaieb L, Antal A, Walsh V, Paulus W (2008). Frequency-dependent electrical stimulation of the visual cortex. Curr Biol.

[28] Kinoshita M, Ikeda A, Begum T, Yamamoto J, Hitomi T, Shibasaki H (2005). Low-frequency repetitive transcranial magnetic stimulation for seizure suppression in patients with extratemporal lobe epilepsy-a pilot study. Seizure.

[29] Misawa S, Kuwabara S, Shibuya K, Mamada K, Hattori T (2005). Low-frequency transcranial magnetic stimulation for epilepsia partialis continua due to cortical dysplasia. J Neurol Sci.

[30] Lefaucheur JP (2006). New insights into the therapeutic potential of non-invasive transcranial cortical stimulation in chronic neuropathic pain. Pain.

[31] Martin JL, Barbanoj MJ, Perez V, Sacristan M (2003). Transcranial magnetic stimulation for the treatment of obsessive-compulsive disorder. Cochrane Database Sys Rev.

[32] Post RM, Kimbrell T, Frye M, George M, McCann U, Little J (1997). Implications of Kindling and Quenching For the Possible Frequency Dependence Of rTMS. CNS Spectrums.

